# Deflationary Extraction Transformer for Speech Separation with Unknown Number of Talkers

**DOI:** 10.3390/s25164905

**Published:** 2025-08-08

**Authors:** Sangwon Lee, Han-Gyu Kim, Gil-Jin Jang

**Affiliations:** 1 School of Electronic and Electrical Engineering, Kyungpook National University, Daegu 41566, Republic of Korea; lsw0767@knu.ac.kr; 2 AI Model, NAVER Cloud, Seongnam 13561, Republic of Korea; hangyu.kim@navercorp.com

**Keywords:** speech separation, speaker diarization, SepFormer, permutation-invariant training, Conv-TasNet

## Abstract

Most speech separation techniques require knowing the number of talkers mixed in an input, which is not always available in real situations. To address this problem, we present a novel speech separation method that automatically finds the number of talkers in input mixture recordings. The proposed method extracts the voices of individual talkers one by one in a deflationary manner and stops the extraction sequence when a predefined termination criterion is satisfied. The backbone separation model is built based on the transformer architecture with permutation-invariant training to avoid ambiguity in identifying talkers at the output. The experimental results on the Libri5Mix and Libri10Mix datasets show that the proposed method without the number of talkers as input significantly outperforms state-of-the-art models that are provided with the number of talkers.

## 1. Introduction

Human auditory systems often show a remarkable ability to focus on a specific acoustic target in a noisy environment with background noise and interfering speech, commonly known as the Cocktail Party Effect [1,2]. However, for a machine, it is considered a challenging task to emphasize or separate a specific acoustic source from a single-channel noisy mixture. The task can be classified into either speech separation, where the voices of all talkers are required, or speaker extraction, where only a subset of talkers is needed by applications. One of the fundamental problems associated with speech separation is permutation ambiguities, i.e., often, the separated output signals are not matched with their correct target sources. One solution is utterance-level permutation-invariant training (uPIT) [3], which is widely adopted by many conventional speech separation techniques [4,5,6,7].

The other challenge in the speech separation task is ascertaining the number of speakers talking simultaneously in the input recordings. Since human speakers are not always talking, this number changes over time, and the separation method should track those instantaneous changes to achieve decent separation performance. The conventional systems often employ an architecture with a fixed number of channels at the output, so the trained models are not able to cope in cases where the number of talkers is varying or unknown, leading to failure in separating individual voices due to the unexpected number of talkers in a particular recording. Speaker extraction systems, however, are free from permutation ambiguity since they only estimate one speech signal at a time from a given mixture. To achieve successful extraction, reference data indicating the target talker is required, such as a sample utterance, speaker embedding vectors, or temporally aligned video recordings of the target talker [8,9,10,11]. These methods require speaker enrollment via a small number of training samples, so their use is limited to specific applications where the speaker information is available in advance.

To tackle the problem of resolving the number of talkers in an input recording, we introduce a novel speech separation method based on a sequential speech extraction approach. This proposed method generates the estimates of speech signals for all the clean sources from a given mixture by repeatedly applying speech extraction in a deflationary manner. The speech extraction block adopted by the proposed method separates the input into a target source and residual signal, and the residual signal is used as the mixed input in the next sequence. The number of sources is estimated by the number of extraction steps. We also propose a sequence termination criterion to stop the sequential extraction steps, which enables determining the optimal estimate regarding the number of sources in the mixture. The basic speech extraction network that splits the input mixture into a target and a residual is constructed via combining transformer modules inspired by SepFormer [7], and one-and-rest permutation-invariant training (OR-PIT) [12] is used to construct and train the sequential speech separation architecture that recursively executes the extraction blocks. Finally, the proposed sequence termination criterion finds the number of sources, which is not provided as an input.

The rest of this paper is organized as follows. Section 2 describes the conventional speech separation techniques, and Section 3 explains the proposed deflation-based speech separation method and the sequence termination criterion. Section 4 presents the experimental setup and outlines the results before providing a detailed analysis. Finally, Section 5 summarizes the contributions of this paper.

## 2. Conventional Speech Separation

### 2.1. Problem Formulation

The goal of speech separation is to recover all of the clean speech signals from a given mixture. It is assumed that there are multiple talkers speaking simultaneously, and their voices are picked up by a single microphone. The recorded signal is a simple addition of speech signals of the assumed multiple talkers. If we represent the recording via a multi-dimensional vector of length *T*, $m\in R^{T}$, it is expressed by

(1) $$m=\sum_{c=1}^{C}s^{\left(c\right)}\;,$$

where $s^{\left(c\right)}\in R^{T}$ is the clean speech of talker *c*, and *C* is the number of talkers. The speech separation task is equivalent to recovering the set of the original clean speech signals. The separation function, typically now implemented via a neural network, generates a set $\hat{S}$, given the mixture recording m in Equation (1):

(2) $$\hat{S}=\left\{{\hat{s}}^{\left(1\right)},\ldots ,{\hat{s}}^{\left(C\right)}\right\}=N\left(m\right)\;,$$

where ${\hat{s}}^{\left(c\right)}$ is the estimate of the clean speech $s^{\left(c\right)}$ of talker *c*, and N(·) is the separation network. The separation methods are characterized by the selection of N(·).

Many recent speech separation techniques are based on transformers [13]. One of the conventional methods, TaSNet [4], was constructed by inserting a separation block between the encoder and the decoder of a pretrained transformer network. The separation block estimates source masks to isolate individual speaker features at the encoder output map and reconstructs separated speeches by the decoder. Conv-TasNet [5] applied temporal convolutional networks to improve the performance of TaSNet. SuDoRMRF [6] has also utilized the encoder–decoder architecture of the transformer and applied U-shaped convolution blocks to the separation module between the encoder and the decoder. Among transformer-based models, SepFormer [7] is another masking-based separation method that is known to present state-of-the-art results on 2mix and 3mix speech separation tasks [14].

### 2.2. One-And-Rest Permutation-Invariant Training (OR-PIT)

**Figure 1:**
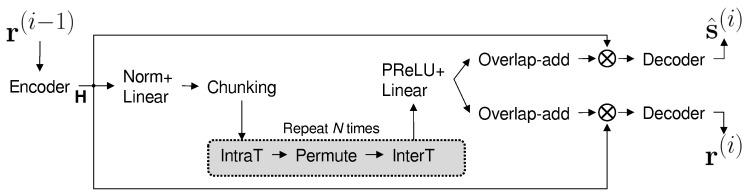
The architecture of the masker network in OR-PIT ($N_{\mathrm{ORPIT}}$). Operator ⊗ represents element-wise multiplication.

The SepFormer and its variants basically have a fixed number of output channels. If there is a change in the number of the source speakers, the number of maskers should also be changed and a new number of maskers have to be built from scratch. This becomes more critical when the number of talkers is completely unknown. To handle cases where the number of talkers is unknown or not fixed, OR-PIT [12] was applied to the SepFormer network. OR-PIT solves the problem by filtering out sources one by one, i.e., it extracts one source and returns the mixture of the others by the remainder of the extraction. The extraction step is carried out until the desired number of sources is extracted. It can be described by the following recursive formula:

(3) $$\begin{array}{ccc} {\hat{s}}^{\left(i\right)},\;r^{\left(i\right)} & = & N_{\mathrm{ORPIT}}\left(r^{\left(i-1\right)}\right)\;, \end{array}$$

where *i* is the iteration index, and ${\hat{s}}^{\left(i\right)}$ and $r^{\left(i\right)}$ are the extracted source and the residual at iteration *i*, respectively. The OR-PIT separation network, $N_{\mathrm{ORPIT}}$, extracts ${\hat{s}}^{\left(i\right)}$ from the previous residual signal $r^{\left(i-1\right)}$. The detailed architecture of $N_{\mathrm{ORPIT}}$ is shown in Figure 1. The mixture m in Equation (1) is given to the encoder of a pretrained transformer network, generating a feature map $H\in R^{F\times T^{\prime }}$. *F* is the dimension of the latent representation generated by the encoder, and $T^{\prime }=\left(2T/L\right)-1$, where *L* is the encoding compression factor [15]. The feature map is then passed to a masker net, which is constructed by a sequence of intra-transformer, permutation-invariant learning, and inter-transformer, denoted by IntraT, Permute, and InterT, respectively. The output of the maker net is a multi-dimensional map with a size that is identical to the size of the encoder output H so that the masked feature map can be generated for each of the target speakers. A decoder finally generates ${\hat{s}}^{\left(C\right)}\in R^{T}$ from the masked feature map at the final step. In Equation (3), the initial residual starts from the mixture of all the sources, i.e., $r^{\left(0\right)}=m$. After the last source is extracted, ideally, no residual signal is expected to remain. Therefore, the final residual of the recursive formula in Equation (3) is $r^{\left(C\right)}\approx 0$, where *C* is the number of mixed sources.

The order of sources to be extracted is hard to determine, so OR-PIT uses an objective function that leads ${\hat{s}}^{\left(i\right)}$ to target a specific speech source and the residual $r^{\left(i\right)}$ to target all sums of the non-target sources. Specifically, the objective function at step *i* is defined as follows [12]:

(4) $$J\left(i\right)=\max_{c}\left[g\left({\hat{s}}^{\left(i\right)},s^{\left(c\right)}\right)+\frac{1}{C-1}g\left(r,\sum_{k\neq c}s^{\left(k\right)}\right)\right]\;,$$

where $g(\hat{s},s)$ is a function for measuring the similarity between the estimated source $\hat{s}$ and the target s. In OR-PIT, it is defined by the scale-invariant signal-to-distortion ratio (SI-SDR), which has been adopted in many speech separation methods [4,5,16]. SI-SDR, also denoted as SI-SNR (scale-invariant signal-to-noise ratio), is computed by the following [12]:

(5) $$\begin{array}{ccc} s_{target} & = & \frac{\langle \hat{s},s\rangle s}{{∥s∥}^{2}}\;,\;e_{noise}=\hat{s}-s_{target}\;,\\ g\left(\hat{s},s\right) & = & \mathrm{SI}-\mathrm{SDR}\left(\hat{s},s\right)=10\log_{10}\frac{∥s_{target}{∥}^{2}}{∥e_{noise}{∥}^{2}}\;, \end{array}$$

where $s_{target}$ is the target with its scale adjusted to the estimated source, and $e_{noise}$ is the scale-matched noise signal between the estimated source and the target. SI-SDR is one of the scale-invariant error measures used to prevent the model’s only manipulation of the gain of the estimated signal since there are some points relative to which only scaling could improve the SNR. In Equation (4), the "max" operation ensures that the estimated source should be matched to the closest source at every step, and the network weights are updated to maximize the objective function J(i). In the training phase, the number of sources *C* is known when computing Equation (4), because the training dataset includes all the labels of the source speakers. However, the number of speakers is not always known for real recordings. For recursive separation techniques such as OR-PIT, only a binary decision on when to stop is necessary instead of inferring the correct value of *C*. The binary classifier for the recursion termination is implemented by a two-class deep neural network in OR-PIT [12] or a simple energy-based criterion in uPIT [3] and dual-path recurrent neural network [17].

### 2.3. SepFormer-Based Separation Block Design

**Figure 2:**
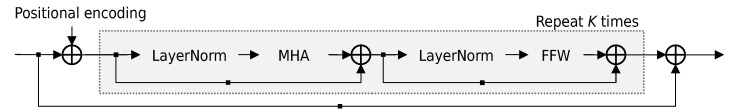
Dual-path implementation of IntraT and InterT modules for the separation block of SepFormer and OR-PIT.

The separation block generates a masker in the feature map domain, and it consists of IntraT, Permute, and InterT modules between the encoder–decoder pipeline, as shown in Figure 1. Both IntraT and IterT employ masking-based dual-path architectures [17], and their implementation is shown in Figure 2. The first path consists of a normalization layer and a multihead attention layer, and a masked encoder map is generated by multiplying attention relative to the encoder output. The second path consists of another normalization layer followed by a feedforward layer. The IntraT-Permute-InterT sequence is repeated a given number of times (*K* in Figure 2) to refine the separation masker map. The separation block in a dual-path approach improves the efficiency of memory usage in transformers and enables learning both short- and long-term dependencies.

## 3. Proposed Method

### 3.1. Sequential Speech Separation via Deflationary Extraction

The OR-PIT network, $N_{\mathrm{ORPIT}}$ in Equation (3), infers an estimated source and its residual at the same time, where the residual is a mixture of all other non-target sources. The input is the residual from the previous recursion step so that the same source should not be obtained again. Therefore, $N_{\mathrm{ORPIT}}$ is expected to double the information of the input, often leading to unstable and unpredictable separation results. We remove the residual estimation at the output to utilize the full capacity of the network and to focus on the source extraction only. The input of the next recursion is obtained by subtracting the estimated source from the input of the current step. Let the initial input mixture be $r^{\left(0\right)}=m$; then, the deflationary extraction process can be described with the iteration index *i* as follows:

(6) $${\hat{s}}^{\left(i\right)}=N_{\mathrm{DEX}}\left(r^{\left(i-1\right)}\right)\;,\;r^{\left(0\right)}=m\;,$$

where i∈{1,…,C} is the recursion index, ${\hat{s}}^{\left(i\right)}$ is the extracted source at iteration *i*, and $N_{\mathrm{DEX}}$ is the proposed deflationary extraction network. The outputs of $N_{\mathrm{ORPIT}}$ in Equation (3) are the target source ${\hat{s}}^{\left(i\right)}$ and the residual $r^{\left(i\right)}$, whereas the output of $N_{\mathrm{DEX}}$ is ${\hat{s}}^{\left(i\right)}$ only. The number of parameters and the computational overhead of $N_{\mathrm{DEX}}$ can be reduced roughly by half compared to $N_{\mathrm{ORPIT}}$. The estimated residual at step *i*, $r^{\left(i\right)}$, is obtained by subtracting the extracted source from the residual at step i−1,

(7) $$r^{\left(i\right)}=r^{\left(i-1\right)}-{\hat{s}}^{\left(i\right)}\;.$$

**Figure 3:**
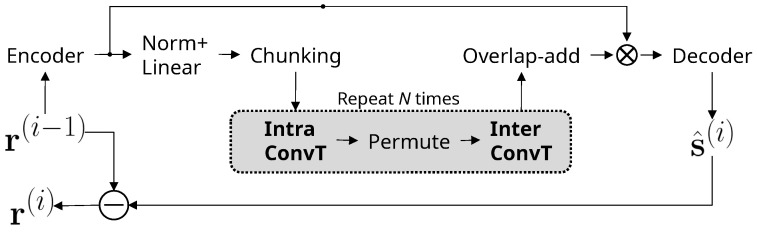
The architecture of the masker network of the proposed method ($N_{\mathrm{DEX}}$). Operator ⊖ represents element-wise subtraction.

Figure 3 shows a single iteration step of the proposed method. Compared to Figure 1, the output is the estimated source ${\hat{s}}^{\left(i\right)}$ only, and the residual of the current extraction step, $r^{\left(i\right)}$, is obtained by subtracting the estimated source from $r^{\left(i-1\right)}$ instead of direct estimation. The intra- and inter-transformers of the separation block are implemented by convolutional transformers. At the output, only the estimated target, ${\hat{s}}^{\left(i\right)}$, is generated by the decoder. The residual of the separation is obtained by subtracting the target from the input, $r^{\left(i-1\right)}$, to reduce the overhead of estimation. We removed the PReLU+Linear layer, which plays the role of a mask multiplicator and a source splitter in SepFormer. The benefit of residual extraction by subtraction is that the target source is less likely to remain in the residual, so failure in splitting the target source and its residual can be avoided more effectively.

### 3.2. Source Extraction Block Design

**Figure 4:**
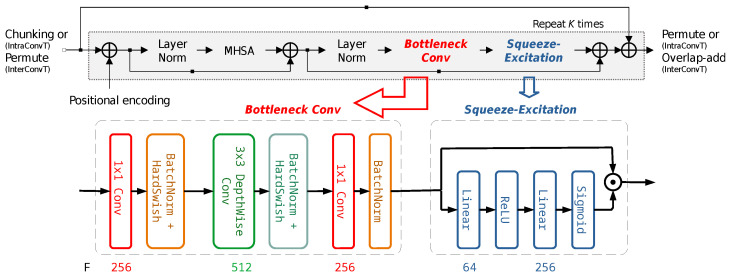
Dual-path implementation of IntraConvT and InterConvT in the separation block of the proposed deflationary extraction approach.

We propose a modified architecture for the source extraction block that is best suited for single-source extraction. Convolutional layers are added to IntraT and InterT, and we denote them as IntraConvT and InterConvT in Figure 3. Their detailed implementation is shown in Figure 4. In the proposed IntraConvT and InterConvT, a multihead self-attention module generates a masked encoder map, which is then passed to the normalization and bottleneck convolution layers, and a squeeze-excitation module elaborates the masked encoder map. The module sequence is repeated a fixed number of times (*K*). The deep feedforward layer is replaced with parameter-efficient convolution blocks inside of every transformer layer. We employ MobileNetV2-style [18,19] bottleneck convolutions that consist of a sequence of 1×1, 3×3, and 1×1 convolution layers. Batch normalization [20] and the HardSwish activation [21] function are applied to all the convolution layers except the last layer. After the bottleneck convolution, we applied a squeeze-and-excitation block [22]. The bottleneck convolution layer reorganizes the channels of the output feature map in such a way that independent sources are split and placed on different channels, and the squeeze-and-excitation block is learned to choose a subset of channels of the target source components only. The whole separation module is repeatedly learned several times (*K* in Figure 4) for the extraction to be more accurate. The number of parameters of the deep feedforward network in IntraT and InterT (Figure 2) is roughly $8F^{2}$, where *F* is the feature map size. However, in the case of the proposed IntraConvT and InterConvT (Figure 4), it is F(F/2+20). For example, if F=256, replacing the deep feedforward layer in the proposed convolution block saves up to 93% in the number of parameters. We adopted this bottleneck–squeeze-excitation architecture because it is better suited for single-source extraction, while the separation block of SepFormer and OR-PIT shown in Figure 1 is required to retain the residual information as well.

As shown in Equation (4), the conventional uPIT [3] computes similarity using g(·,·) between the network output and the target of all *C* speakers, requiring a total of C!=C(C−1)⋯2·1 pairs. In our work, however, it only computes *C* pairs since the proposed network $N_{\mathrm{DEX}}$ only generates a single estimated signal $\hat{s}$. Therefore, the objective function is simplified as

(8) $$J\left(i\right)=\max_{c}g\left({\hat{s}}^{\left(i\right)},s^{\left(c\right)}\right)\;,$$

where *g* is a similarity metric between the estimated source ${\hat{s}}^{\left(i\right)}$ and target candidate $s^{\left(c\right)}$. The signal similarity is obtained by SI-SDR in OR-PIT, as shown in Equation (5). However, the scale-invariant characteristic is not well suited for the proposed deflationary extraction because r should be obtained by simple subtraction. If the scale of $\hat{s}$ is different from one of the corresponding targets, $\hat{s}$ is not removed effectively, and then, r contains a huge error, which could be accumulated over training iterations, leading to the cumulative degradation of separation performance. To address this problem, we use simple SNR for *g* to force the model to keep the scale of $\hat{s}$ at the same level with one of the corresponding targets:

(9) $$\begin{array}{ccc} g\left(\hat{s},s\right) & = & \mathrm{SNR}\left(\hat{s},s\right)=10\log_{10}\frac{{∥s∥}^{2}}{∥\hat{s}{-s∥}^{2}}. \end{array}$$

Another advantage of using simple SNR is that it may reduce the permutation ambiguity because there is no additional scaling mismatch between the estimated and the target sources, when compared to other separation methods that use scale-invariant objective functions [4,5,12,16].

### 3.3. Sequence Termination Criterion

**Figure 5:**
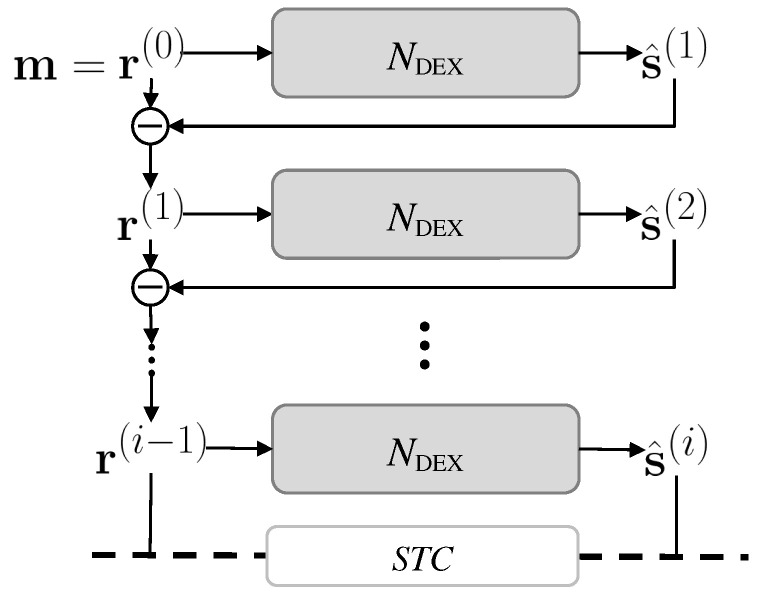
The complete pipeline of the proposed method with a sequence termination criterion.

In most real cases, the number of speakers is usually unknown, so the recursive extraction model should automatically figure out the number of iterations for sequential speaker extraction. In contrast to the trainable binary classifier of OR-PIT [12], we define a sequence termination criterion (STC) without additional parameters to learn. We designed an algorithm to quit the deflationary extraction with a simple thresholding technique. The criterion simply compares the average powers of an estimated signal ${\hat{s}}^{\left(i\right)}$ and its residual signal $r^{\left(i\right)}$ to the predefined threshold values $H_{s}$ and $H_{r}$, respectively. The deflationary extraction sequence continues until one of the average powers becomes smaller than the threshold. This simple STC works well since we force the model to match the power of an estimated signal ${\hat{s}}^{\left(i\right)}$ with the power of corresponding target source $s^{\left(c\right)}$ by employing SNR as the loss function instead of SI-SDR. Algorithm 1 describes the proposed sequential speech separation with a termination criterion. At every iteration, the powers of both ${\hat{s}}^{\left(i\right)}$ and $r^{\left(i\right)}$ are computed and compared to thresholds $H_{s}$ and $H_{r}$, respectively. The extraction is terminated when either the extracted signal or the residual falls below their corresponding threshold values. Figure 5 illustrates the whole pipeline of the proposed method with STC. It begins with the mixture of all speakers, $r^{\left(0\right)}=m$, and extracts the speech of a single speaker at every iteration. The extraction is terminated with STC.
**Algorithm 1** Sequential speech separation**Require:** estimation power threshold $H_{s}$, residual power threshold $H_{r}$ 1:i←1 2:$r^{\left(0\right)}\leftarrow m$ 3:$P_{s}\leftarrow \infty$ 4:$P_{r}\leftarrow \infty$ 5:**while** $P_{s}>H_{s}$ & $P_{r}>H_{r}$ **do** 6:     ${\hat{s}}^{\left(i\right)}\leftarrow N_{\mathrm{DEX}}\left(r^{\left(i-1\right)}\right)$ 7:     $r^{\left(i\right)}\leftarrow r^{\left(i-1\right)}-{\hat{s}}^{\left(i\right)}$ 8:     $P_{s}\leftarrow \frac{1}{T}\sum_{t}{\left({\hat{s}}_{t}^{\left(i\right)}\right)}^{2}$ 9:     $P_{r}\leftarrow \frac{1}{T}\sum_{t}{\left({\hat{r}}_{t}^{\left(i\right)}\right)}^{2}$10:   i←i+111:**end while**

## 4. Evaluation

In our experiments, we adopted LibriMix [14], which is one of the popular benchmark datasets used for speech separation tasks. LibriMix is generated by mixing the audio files in LibriSpeech [23]. Among LibriSpeech subsets, we used the 8 kHz version of train-clean-360, dev-clean, and test-clean to generate training, validation, and test sets, respectively. The original script [14] only provides “2mix” and "3mix" subsets, where two and three are the numbers of speakers. We modified the script to generate 5mix and 10mix subsets as well.

### 4.1. Training and Evaluation Details

We used 256 convolution filters, with a kernel size of 16 and stride of 8 samples. The masker network processes a chunk of size 100 with 50% overlap. We used stacking sizes N=3 for SepFormer blocks and K=8 for IntraT and InterT each, totaling 48 transformer encoders. The expansion ratio is 2, generating 512 hidden channels in convolution transformers. We used a batch size of 6, gradient clipping to limit the $l^{2}$ norm of the gradients to 5, and automatic mixed precision powered by PyTorch 2.0, Automatic Mixed Precision package (The Linux Foundation, San Francisco, CA, USA). We used a varied number of speakers with dynamic mixing [24] to train the model. We randomly chose 2 to 5 sources from LibriSpeech audio files to generate 2mix, 3mix, 4mix, and 5mix samples. A single model is trained by audio samples, with the number of talkers varying from 2 to 5. When adding the audio files, the scales of the files were adjusted so that their SNRs should uniformly be distributed in [0 dB, 5 dB], with random speed perturbation uniformly carried out in [0.95, 1.05]. All actual values of the hyperparameters are listed in Table 1.

**Figure 6:**
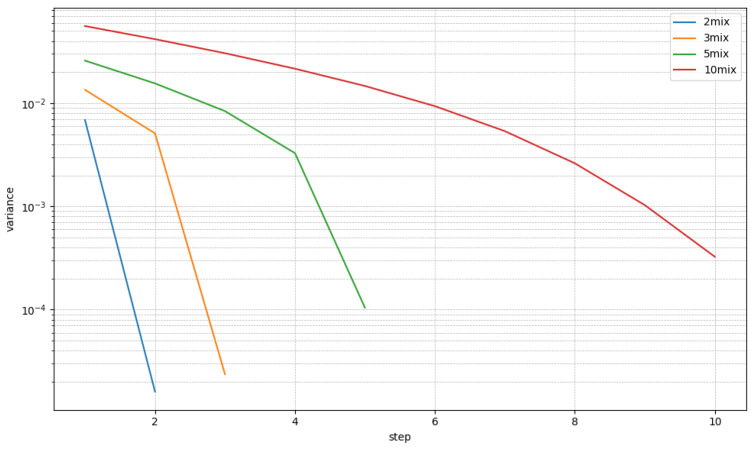
Change in residual variance graph.

For evaluation, we generated 3000 samples for each of the 2mix, 3mix, 5mix, and 10mix cases. The separation experiments are carried out under two conditions, denoted as a known or unknown number of speakers. The known condition means that the true speaker count is given with each of the evaluation samples. In the unknown condition, the true speaker count is not given, and the model should be able to cope with these types of cases. If the model supports fixed speaker counts, known condition performances are reported only. For known condition cases in the proposed method, the sequential step is repeated as many times as the given number of speakers, whereas for unknown condition cases, the extraction step is repeated until the stopping criterion in Algorithm 1 stops the iteration. Both $H_{s}$ and $H_{r}$ threshold values are set to $10^{-4}$. Figure 6 shows the trajectory of the residual signal powers. The x-axis is the number of steps in recursive source extraction, and the y-axis is the mean squared power of residual signals. There are four lines for 2mix, 3mix, 4mix, and 5mix LibriMix samples. The mean squared power of residual signals is computed by $P_{r}=\frac{1}{T}\sum_{t}{\left({\hat{r}}_{t}^{\left(i\right)}\right)}^{2}$, as shown in line 9 of Algorithm 1. The values plotted on the graph are $P_{r}$-averaged over test samples. For 2/3/5mix, it falls below the threshold $H_{r}=10^{-4}$ at steps 2, 3, and 5, respectively. For 10mix, it is above $H_{r}$, because 10mix mixtures are not included in the training samples. However, it is close to $H_{r}$, so most of the samples are successfully predicted.

Most speech separation methods do not guarantee optimal permutation. Therefore, the SI-SDR improvements are measured by matching the indexes of the extracted sources and true sources of the evaluation set. In the sequential extraction methods the extracted source at the current step is excluded from the next step. In unknown conditions, we give some penalties to under- or over-extraction cases by adding or replacing the SI-SDR improvements for wrongly estimated signals with 0, where under-extraction means that the model generates a smaller number of signals than the target and over-extraction means the opposite.

### 4.2. Speech Separation Results

Table 2 shows the evaluation results with a known number of speakers. We have compared the proposed method with various conventional speech separation methods: fully convolutional time-domain audio separation network (Conv-TasNet) [5,14], separation transformer (SepFormer) [7], SepFormer with a pretrained diffusion model (SepFormer + DiffWave) [25], optimal permutation-invariant training (HungarianPIT) [26], iterative separation (SepIt) [27], dual-path recurrent neural network (DPRNN) [17], and one-and-rest permutation-invariant training (OR-PIT) [12]. The SI-SDR improvements in Table 2 are from the original papers on the same LibriMix dataset. In the second column, single task means that the number of speakers is fixed in the model, and the model should be trained with a fixed number of speakers as well. The training column shows the training data types required by each method. The separation performances are measured by SI-SDR improvements in dB units on Libri-nMix datasets, with a varying number of speakers. The number of speakers is given in both training and evaluation phases. Conv-TasNet and SepFormer is trained by 2mix and 3mix, so experimental results are available with 2mix and 3mix only. SepFormer+DiffWave shows the best 2mix result among all methods, but there are no 3mix, 5mix, and 10mix results because the model is configured to support 2mix only. HungarianPIT and SepIt provide 5mix and 10mix results, and DPRNN has 2/3/5mix results. Among single-task methods, the best 2mix result is 21.5 dB of SepFormer + DiffWave, 18.7 of SepFormer for 3mix, and 13.7 dB and 8.2 of SepIt for 5mix and 10mix. There is no single method that is applicable to all numbers of speaker conditions. The multi-task separation methods can deal with varying numbers of speakers using a single model. We implemented OR-PIT [12] with minimum modifications to support a varying number of mixtures. It is trained by the 2/3/4/5mix dataset, and it provides separation results for the 2/3/5/10mix evaluation set. Although no 10mix training set is given, the trained model can be applied to the 10mix evaluation set. Compared to single-task methods, the SI-SDR results are 4.3 dB and 2.2 dB lower for 2mix and 3mix and 0.4 dB and 0.2 dB higher for 5mix and 10mix. The proposed method, deflationary extraction transformer (DExFormer in Table 2), also supports multi-tasks. A single DExFormer model is trained by the 2/3/4/5mix dataset, and the separation results for the 2/3/5/10mix evaluation sets are obtained. For 2mix and 3mix cases, the SI-SDR improvements are 3.2 dB and 1.0 dB lower than SepFormer and SepFormer + DiffWave because the proposed method is optimized for general and realistic cases. Instead, the proposed method outperforms SepIt for 5mix and 10mix cases, with values being 2.2 dB and 1.5 dB higher. Given the number of speakers, the proposed method mostly outperforms the conventional methods, especially when there are more than three speakers. OR-PIT and the proposed method extract a single speaker one by one, so the extraction errors are unavoidably accumulated. Therefore, their performances gradually drops as the number of speakers grow. In contrast, other single-task methods use different models for different numbers of speakers, so no error accumulation is expected. Comparing 5mix SI-SDR improvements, HungarianPIT, SepIt, and DPRNN show 12.7, 13.7, and 8.7, respectively, while the extraction-based methods, OR-PIT, and the proposed method show 14.1 and 15.9, respectively. Similar observations can be found with 10mix cases, although the improvements are smaller than the 5mix ones. These improvements are due to the following explanations:

- The multi-task methods share the same separation/extraction module for all numbers of speakers, and the shared module acts like a pretrained model in training, with mixtures of a higher number of speakers.
- The benefit of model sharing is much higher than the error accumulation, which is proven by carrying out comparisons with the single-task methods in 5mix and 10mix cases.

Moreover, the performance achieved on the 10mix case is impressive since 10mix mixture samples are not included in the training dataset.

Table 3 shows the evaluation results with an unknown number of speakers. We have chosen gated LSTM [15] and gated LSTM with a pretrained diffusion model [25] as the state-of-the-art methods for multiple-speaker separation. Since the numbers of speakers are unknown, only multi-task models are applicable. The separation performances are measured by SI-SDR improvements in dB units on Libri-nMix evaluation datasets, with a varying number of speakers. The number of speakers is given in training and not given in the evaluation phase. We added sequence a termination criterion module to OR-PIT (OR-PIT + STC) and the proposed DExFormer (DExFormer + STC). There is only one model for each OR-PIT + STC and DExFormer + STC trained by the 2/3/4/5mix dataset, and separation results for the 2/3/5/10mix evaluation sets are obtained. In terms of SI-SDR improvements, DExFormer outperforms OR-PIT with SepFormer block by 1.0 dB, 1.3 dB, 1.8 dB, and 1.4 dB for the 2/3/5/10mix evaluation sets, respectively.

Based on known and unknown experimental results, the proposed DExFormer architecture is shown to improve the separation performance from 1.0 dB to 1.8 dB over SepFormer with OR-PIT. Comparing the results of DExFormer with varying number of speakers in Table 3, the proposed DExFormer showed very little performance degradation except the 10mix. The evaluation results of gated LSTM models are provided for 5mix and 10mix only in their original papers. The SI-SDR improvements are 1.5 dB higher for 5mix evaluation and 0.1 dB lower for 10mix than that of gated LSTM + DiffWave. Even in conditions without the given number of speakers, the proposed method still outperforms conventional state-of-the-art methods. Comparing Table 2 and Table 3, the performance drop is up to 0.2 dB in 2/3/5mix. For 10mix, it is 0.8 dB, because more errors are expected with a larger number of speakers in the mixed recordings. Overcoming this degradation problem in many speakers speech separation could become a future research direction.

### 4.3. Analysis of the Proposed Sequence Termination Criterion

For unknown cases, predicting the true number of speakers is crucial to successful speaker separation. We have carried out comprehensive analyses on the prediction results of the proposed STC. For each evaluation sample, the predicted speaker count is obtained by the number of iterations before the termination of the extraction sequence, and then, it is compared to the true count. Table 4 shows a confusion matrix for the prediction of speaker counts. Each column lists the number of training samples with their true speaker counts given by digits 2 to 5, and each row lists the number of samples with predicted speaker counts with digits 2 to 6. The diagonal elements are the number of samples with correct prediction results, and the off-diagonal ones are the count of samples with incorrect predictions. There are no samples with a true count of 6 because only the 2/3/4/5mix datasets are used in training, so the elements in row 6 are all incorrect.

From the confusion matrix, we have computed the precision and recall rates for each of the cases. The precision rate is calculated by $\frac{TP}{TP+FP}$, where TP and FP stand for the numbers of true positives and false positives, respectively. The computed precision rates are shown in the last column of Table 4. From 2 to 5 speakers, they are 99.8%, 98.0%, 95.6%, and 98.2%, respectively. In Table 4, 200 samples from five speakers are predicted as six speakers, and they are all false positives; the number of true positives is 0 because there is no sample with six speakers. Therefore, the precision of six speakers is $\frac{0}{0+200}=0$. This shows that the proposed STC algorithm predicts speaker counts effectively in most cases. Though there is more than a 2% drop in precision rate at four speakers, less than 5% of errors is observed. The recall rate is defined by $\frac{TP}{TP+FN}$, where FN stands for the number of false negatives. The computed recall rates are, from 2 to 5 speakers, 99.7%, 99.1%, 96.8%, and 89.4%, respectively. There are no samples with true speaker counts being six, so both TP and FN are 0, and it is impossible to compute recall in this case. F1 scores are computed by 2(precision×recall)/(precision+recall), and they are listed in the bottom row. The highest score of 99.7% is achieved for two speakers, and the lowest score of 93.6% is achieved for five speakers.

We have compared the proposed STC with other methods. Table 5 shows the accuracy of binary decisions for speech or noise, and it shows multi-class classification accuracy values for speaker counts. The results are provided by OR-PIT [12]. The binary classifier is an AlexNet-like convolutional neural network [28]. If the classification’s output is noise, this implies that no speech remains, and OR-PIT stops recursion in Equation (3). The multi-class classifier is also implemented by AlexNet, and it determines how many speakers are mixed. Its accuracy is much lower than that of the binary classifier. The role of the binary classifier is identical to the STC of the proposed method. Its confusion matrix is not given [12], but an accuracy of 95.7% on the WSJ0-2mix and WSJ0-3mix datasets [29] is reported. Although one-to-one comparison is not feasible, the proposed STC shows F1 scores of 93.6% to 99.7% on the LibriMix dataset, as shown in Table 4, so its performance is on par with the binary classifier of OR-PIT. Table 6 shows the results of speaker count detection with DPRNN [17] and Gated LSTM [15]. Speaker count detection experiments are carried out on the WSJ0-2/3/4/5mix datasets [15,29]. The experimental results are given by the confusion matrices in percentages only, and sample counts are not available. For a mixed sample, the average power of each output channel is computed and verified if the computed power is above a predefined, fixed threshold. The speaker count is determined by the number of channels, with their powers being above the threshold. The confusion matrices in Table 6 are obtained by comparing a true number of speakers and the predicted speaker count for each of the samples. Diagonal elements of the confusion matrices are the ratios of correct sample counts to the total number of samples, which are identical to recall rates. Recall rates are 81.3%, 64.4%, 46.2%, and 85.6% for DPRNN; 84.6%, 69.0%, 47.5%, and 92.3% for Gated LSTM; and 99.7%, 99.1%, 96.8%, and 89.4% for the proposed DExFormer, as shown in Table 4. Therefore, the prediction accuracy of the proposed method is shown to be much higher than those of DPRNN and Gated LSTM. In Table 6, there are more errors in the lower triangular part, where the predicted speaker counts are larger than the actual number of speakers. For example, the percentage of predicting five for samples with a true speaker count of four is 40.7% for DPRNN and 43.9% for Gated LSTM. The separation modules are trained in a way that if the predicted speaker count is higher than the actual number of speakers, silent outputs should be generated for the extra channels [15]. Thus, low prediction accuracies do not have significant effect on the output of SI-SDR.

In summary, the proposed STC and speaker separation has very high accuracy in predicting the number of speakers compared to other methods. It requires two learnable threshold values for speech and residual signals, which are trained together with speech separation parameters. OR-PIT [12] uses a binary classifier implemented by convolutional neural networks [28], and our STC achieved similar prediction performances compared to the complex OR-PIT model. Gated LSTM [15] also adopted a simple thresholding detector but without learning, and our proposed method showed much higher prediction accuracies. In spite of the huge difference in prediction accuracies, the difference in SI-SDR improvements shown in Table 3 is relatively small, because Gated LSTM does not require the exact number of speakers when separating multiple speeches. However, for applications where the exact speaker counts is required, the proposed STC should be beneficial.

## 5. Conclusions

In this paper, we proposed a speech separation system for many-speaker speech separation based on a sequential speech extraction approach and an efficient separation network used for this system. Also, we defined an STC that makes this system applicable for cases with an unknown number of speakers. Our method improved the scale-invariant SDR improvement, which is a common metric for speech separation tasks, achieving a new state-of-the-art performance for the Libri5Mix and Libri10Mix datasets with and without prior knowledge of the number of speakers. The proposed method enables automatic speech recognition in cases where multiple speakers are talking simultaneously. It requires recordings collected by only a single microphone, so by using the proposed method, the applicability of speech recognition is greatly enhanced because multiple audio sensors are not required. The proposed method is data-driven, so it can be applied to various cases once training data reflect the environment where it should be utilized. It can also be applied to many other modalities, such as music sounds, audio retrieval, brain waves, electromagnetic waves, and many other sensor applications as well.

## Figures and Tables

**Table 1:** Actual values of hyperparameters of the separation modules.

Module	Parameter	Value	Output Shape
Encoder	kernel size	16	$\left[B,\;T^{\prime },\;F\right]$
	stride	8	
	*F*	256	
Linear	*F*	256	$\left[B,\;T^{\prime },\;F\right]$
Chunking	chunk size	100	$\left[B,\;T^{\prime \prime },\;\mathrm{chunk}\;\mathrm{size},\;F\right]$
Masker Network	*N*	3	$\left[B,\;T^{\prime \prime },\;\mathrm{chunk}\;\mathrm{size},\;F\right]$
	*K*	8	
MHSA	heads	8	$\left[B,\;T^{\prime \prime },\;\mathrm{chunk}\;\mathrm{size},\;F\right]$
Bottleneck Conv	expansion ratio	2	$\left[B,\;T^{\prime \prime },\;\mathrm{chunk}\;\mathrm{size},\;F\right]$
	*F*	256	
Squeeze-Excitation	squeeze ratio	0.25	$\left[B,\;T^{\prime \prime },\;\mathrm{chunk}\;\mathrm{size},\;F\right]$
	*F*	256	
Decoder	kernel size	16	$\left[B,\;T\right]$
	stride	8	
	*F*	1	
STC	$H_{s}$	$10^{-4}$	$\left[B,\;T\right]$
	$H_{r}$	$10^{-4}$	
	learning rate	0.0001	

**Table 2:** Experimental results for known number of speakers cases.

Method	Task	Training	2mix	3mix	5mix	10mix
Conv-TasNet [5,14]	single	2/3mix	14.7	12.1	-	-
SepFormer [7]	single	2/3mix	20.6	18.7	-	-
SepFormer + DiffWave [25]	single	2mix	21.5	-	-	-
HungarianPIT [26]	single	5/10mix	-	-	12.7	7.8
SepIt [27]	single	5/10mix	-	-	13.7	8.2
DPRNN [17]	single	2/3/5mix	18.1	14.7	8.7	-
OR-PIT [12] *	multi	2/3/4/5mix	17.2	16.5	14.1	8.4
DExFormer (ours)	multi	2/3/4/5mix	18.3	17.7	15.9	9.7

* Our implementation with minimal modification.

**Table 3:** Experimental results of unknown number of speakers cases.

Method	Task	Training	2mix	3mix	5mix	10mix
Gated LSTM [15]	multi	5/10mix	-	-	12.7	7.7
Gated LSTM + DiffWave [25]	multi	5/10mix	-	-	14.2	9.0
OR-PIT * [12] + STC	multi	2/3/4/5mix	17.2	16.4	13.9	7.5
DExFormer + STC (ours)	multi	2/3/4/5mix	18.2	17.7	15.7	8.9

* Our implementation with minimal modification.

**Table 4:** Confusion matrix in number of samples obtained by speaker count prediction experiments.

		True Speaker Counts				Total	
		2	3	4	5	Counts	Precision
	2	2989	7	0	0	2996	99.8%
Predicted	3	8	2972	47	5	3032	98.0%
speaker	4	0	21	2904	113	3038	95.6%
counts	5	0	0	49	2678	2727	98.2%
	6	0	0	0	200	200	0%
Total counts		2997	3000	3000	2996		
Recall		99.7%	99.1%	96.8%	89.4%		
F1 score		99.7%	98.5%	96.2%	93.6%		

**Table 5:** Test accuracy of speech or noise binary classifier and multi-class speaker count classifier of OR-PIT [12].

Model	Accuracy
Binary classifier	95.7%
Multi-class classifier	77.9%

**Table 6:** Results of automatically selecting the number of speakers for a mixed sample. The confusion matrices in percentage are given for DPRNN [17] and Gated LSTM [15].

		True Number of Speakers				
	Predicted	2	3	4	5	Precision
DPRNN	2	81.3%	7.9%	3.2%	0.7%	87.3%
	3	15.9%	64.4%	9.9%	2.4%	69.5%
	4	0.7%	14.5%	46.2%	11.3%	63.5%
	5	2.1%	13.2%	40.7%	85.6%	60.5%
	Recall	81.3%	64.4%	46.2%	85.6%	
	F1 score	84.2%	66.9%	53.5%	70.9%	
	2	84.6%	3.6%	1.2%	0.3%	94.3%
Gated	3	13.7%	69.0%	7.4%	1.6%	75.2%
LSTM	4	0.5%	18.2%	47.5%	5.8%	66.0%
	5	1.2%	9.2%	43.9%	92.3%	63.0%
	Recall	84.6%	69.0%	47.5%	92.3%	
	F1 score	89.2%	72.0%	55.2%	74.9%	

## Data Availability

Data is contained within the article.

## Abbreviations

The following abbreviations are used in this manuscript:

uPIT	Utterance-level permutation-invariant training;
OR-PIT	One-and-rest permutation-invariant training;
STC	Sequence termination criterion;
SepFormer	Separation transformer;
TaSNet	Time-domain audio separation network;
Conv-TasNet	Convolutional time-domain audio separation network;
SNR	Signal-to-noise ratio;
SI-SDR	Scale-invariant signal-to-distortion ratio;
DPRNN	Dual-path recurrent neural network;
DExFormer	Deflationary extraction transformer.
